# A systematic review of the flood vulnerability using geographic information system

**DOI:** 10.1016/j.heliyon.2022.e09075

**Published:** 2022-03-08

**Authors:** Shiau Wei Chan, Sheikh Kamran Abid, Noralfishah Sulaiman, Umber Nazir, Kamran Azam

**Affiliations:** a Faculty of Technology Management and Business (FPTP), Universiti Tun Hussein Onn Malaysia, 86400 Parit Raja, Batu Pahat, Johor, Malaysia; b Faculty of Social & Administrative Science, Department of Management Sciences, The University of Haripur, Hattar Road Near Swat Chowk Haripur, Khyber Pakhtunkhwa, Pakistan

**Keywords:** Disaster, Flood vulnerability, Geographic information system

## Abstract

The world has faced many disasters in recent years, but flood impacts have gained immense importance and attention due to their adverse effects. More than half of global flood destruction and damages occur in the Asia region, which causes losses of life, damage infrastructure, and creates panic conditions among the communities. To provide a better understanding of flood hazard management, flood vulnerability assessment is the primary objective. In this case, vulnerability is the central construct in flood analysis and assessment. Many researchers have defined different approaches and methods to understand vulnerability assessment and how geographic information systems assess the flood vulnerability and their associated risk. Geographic information systems track and predict the disaster trend and mitigate the risk and damages. This study systematically reviews the methodologies used to measure floods and their vulnerabilities by integrating geographic information system. Articles on flood vulnerability from 2010 to 2020 were selected and reviewed. Through the systematic review methodology of five research engines, the researchers discovered a difference in flood vulnerability assessment tools and techniques that can be bridged by integrating high-resolution data with a multidimensional vulnerability methodology. The study reviewed several vulnerability components and directly examined the shortcomings in flood vulnerability approaches at different levels. The research contributed that the indicator-based approach gives a better understanding of vulnerability assessment. The geographic information system provides an effective environment for mapping and precise analysis to mitigate the flood disaster**.**

## 1 Introduction

Flooding is a natural event that causes widespread destruction, adversely affects daily life and raises vulnerability, including physical, social, economic, and environmental exposure. Flood has been identified as an upward condition of water levels in coastal areas, reservoirs, streams, and canals (Abah and Clement, 2013). About 350 million people in the world are affected by floods. It is also predicted that the flood destruction will be double by the end of 2050. It is one of society's most catastrophic environmental hazards and has drawn many researchers' interest to see it in the light of the growing effects of ecological alteration. The ever-increasing population and the combination of properties in built-up areas also increased flooding potential. In the future, the impact of flooding will increase as the population increases (Walker and Burningham, 2011).

By 2030, the effect on individuals living within 100 km of the coast is projected to be much more significant (Abbas et al., 2009). The latest flood effects have given rise to many questions about climate change and the impact of human activity on the global climate (Adams and Adams, 1984) (Rehman et al., 2019) (Abid et al., 2020). Increased population growth is also expected to rise in flood incidence (Adger, 2006). Due to population increase, the valuable surface was turned into a water-resistant area, resulting in erosion, natural rushing, and flood rise. In recent years, the average loss of flooding has risen to around fifty billion USD dollars on average. Analysis has found that between 2010, 2013, 2014, 2015, 2017, 2018, and 2019 increased cases of flood catastrophes (Adger et al., 2005).

Flood disasters struck numerous people in 2000, 2007, 2014, and 2015. Between 2010 and 2020, almost 3.6 billion inhabitants were inundated, comprising 56 percent of the world's total population. During 2010–2020, about 820,000 people in South and North America alone suffered from flood hazards (Rehman et al., 2019). In the least developed countries, flood disasters created dreadful conditions that caused major human trauma, massive losses to the substructure, life threats, and commercial development.

Over the past decade, Bangladesh, Mozambique, Germany, India, China, Malaysia, and the United States have caused disastrous circumstances and tremendous damage to lives and property (Alias et al., 2020). The tragedy is limited to developed countries and significantly impacts the world's most urbanised and developed nations. In 1988–2000 significant damage and economic losses of USD 3.64 trillion were caused in Central America and Asia due to natural and manmade disasters (Andrade and Szlafsztein, 2018). Andrew Hurricane in America has caused losses of around $ 27 billion (Aroca-Jiménez et al., 2020). Flood damages and their potent effects on people should also be considered when locating the case. Highly populated cities are more likely to suffer from flooding, and the impact is different from the asset (Bajracharya et al., 2021).

In city areas, the water contamination crisis has created higher purification costs and worsened issues with health. Even surfaces suffer more significant floods and extensive damage. A demonstration of data from the Database for Emergencies, Balica et al. (2009) showed that since 2010, 52 countries had suffered USD 2 billion in losses to buildings, cattle, or other crops. In addition, one-year flood damage in Southeast Asia, Africa, north and south America exceeded US$5 billion (Balica et al., 2013) (Abid et al., 2021a, b, c). The major causes of floods in India were unexpected precipitation in the southwestern mountain, increasing tropical storms and depressions, riverbed wilting, the inefficacy of rivers with high discharge. In Indian economy was harmed during the floods of 1980–2010, and the flood during this period was also ranked 2nd highest disaster after deficiency (Andrade and Szlafsztein, 2018) (Rehman et al., 2019).

In 1977 and 1978, the highest deaths in India were reported, with an average population of 3.2 billion, while in 2001, public services received massive damage (Andrade and Szlafsztein, 2018). The causes of human devastation, the outbreak of diverse diseases in the river, damage to crop productivity and infrastructure are root causes of fluvial vulnerability. Moreover, disasters were responsible for 80 percent of deaths in women and infants and are more vulnerable to women and children (Balica et al., 2012). Vulnerability varies from place to place, and the degree of correlation impacts policy implementation directly (Bera and Daněk, 2018). Consequently, identifying the region vulnerable to floods is essential for addressing community vulnerability—another critical aspect of community capacity to deal with the effects of floods (Birkmann et al., 2013).

Both operation and strategic analysis and assessment of floods require spatial and hydrological modelling, risk assessment, model estimation. Time analysis is fundamental in this case, projecting, forecasting, and decision-making with real-time risk analysis. Nowadays, the world is facing both natural and manmade disasters (Andrade and Szlafsztein, 2018) (Garbutt et al., 2015) (Sulaiman et al., 2020a, b). To alleviate the impact of a flood, the discussion on coping with the rapid environmental changes needs a systematic vulnerability technique to reduce the flood risk. However, in the past, the flood assessment was used to support the strategic planning and decision-making process, and still, we are unable to mitigate the impact of the flood. In the current scenario, and both physical and environmental changes require more rigorous approaches and methods to assess the flood. The recent development in the Netherlands is to utilise the probabilistic approach towards mitigating the impact of the flood (Kirby et al., 2019). Combining traditional methods with spatial decision bridges a more powerful tool for flood vulnerability assessment and spatial planning.

To measure vulnerability in terms of socio-economic status, Brooks et al. (2005) has given many aspects and an effective model. Brouwer et al. (2007) looked at flood risk and resilience at the local scale to assess the degree of flood exposure in the Malaysia region and how people have dealt with flood damage. He described the flooding as a significant contributor to poverty and income inequality in Bangladesh. Many researchers have also made substantial attempts to explain the damage to floods (Chang and Baiamonte, 2002) (Cannon, 2004) (Chen et al., 2015) (Chakraborty and Joshi, 2016) (Canevari-Luzardo et al., 2017); (Vazire, 2018) evaluated the flood vulnerability from the perspective of case studies and observed that flood vulnerability is an effect of flood hazard. Different modelling techniques like Hydrologic engineering centre (HEC-RAS) models are customized for getting the flood hazards maps of rivers. These models successfully applied on the river of Columbia, Warsaw, Texas, mid-eastern in Dhaka, and many other flood regions and have been found critical in flood vulnerability assessments (Creach et al., 2016) (Rehman et al., 2019).

GIS-based flood vulnerability evaluations are beneficial for massive areas, although more specific aspects of flood dynamics can be explored by hydrodynamic models (Abid et al., 2021a, b, c). The linear techniques have significant shortcomings over parametric strategies of flood vulnerability evaluation. However, combining these two approaches will effectively interpret vulnerability situations in an area (Dandapat and Panda, 2017). These scholars examined flood assessment in an area as a product of likelihood and penalties (Shivaparasad Sharma et al., 2018). Machine learning-based models with less significantly observed flood vulnerability in Haraz watershed of Iran using hybrid and ensemble models (Andrade and Szlafsztein, 2018). They stressed that the choice of suitable model parameters could seamlessly be practical to assimilate flood susceptibleness. Observing responsibility to flood is an essential part of flood risk analysis.

Different techniques have been used to measure flood vulnerability for a long time (Rehman et al., 2019). Therefore, precise comparative assessment is vital for other dimensions (Andrade and Szlafsztein, 2018). Previous studies indicated various methods used to assess the vulnerability. These methods include the vulnerability curve method, Indicator based method, analytical hierarchy process, mapping method, disaster loss data method, and modelling methods through geographic information system (Ebert et al., 2009) (Musa and Shabu, 2019) (Abid et al., 2021a, b, c). Many researchers and policymakers have widely used the vulnerability indicator-based methodology to assess vulnerability. An indicator-based vulnerability methodology, the logical image, has been adopted to utilise the data to examine the vulnerability. The indicator methods aim to measure the potential risk and their response in the hazard regions. A wide range of vulnerability indicators has been found in the literature (Nasiri et al., 2016). For example, the study of Garbutt et al. (2015) presents an index that utilizes the 42 indicators to assess the flood vulnerability.

**Figure 1:**
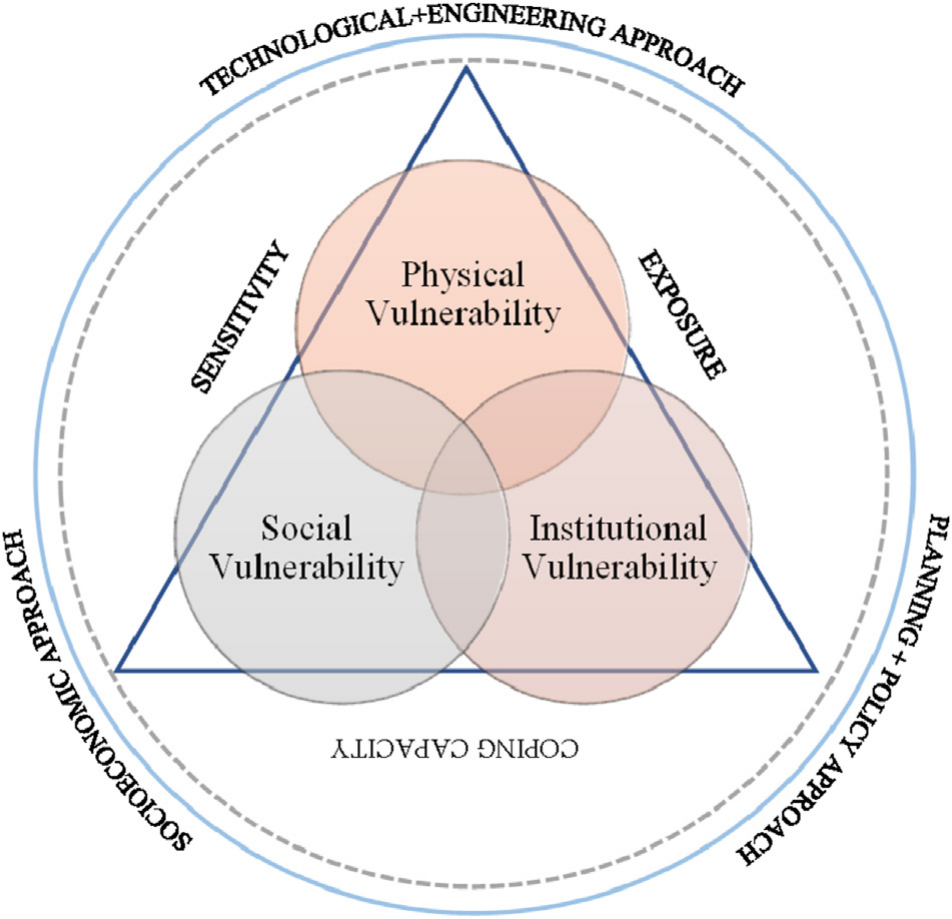
Dimensions of flood vulnerability and flood risk assessment approaches; Modified from Cho and Chang (2017).

This review study tried to reduce the error caused by subjective interpretation in analysing and categorising papers by establishing the conceptual and methodological boundaries of each dimension of flood risk. However, because the definition of vulnerability is still evolving with our understanding of flood possible impacts, and many disciplines use different criteria and methodologies to assess vulnerability in practice, multi-dimensional aspects of urban flood vulnerability can be challenging to categorise, as shown in Figure 1.

This systematic literature review (SLR) paper presents inclusive answers to the following questions regarding the objective of this research.

What are the dimensions of flood vulnerability, and how geographical information is used to assess the flood vulnerability?

1. What are the different methodologies and approaches used in previous studies to address flood vulnerability?
2. How community benefit from these methodologies, techniques, and approaches in disaster?

## 2 Materials and methods

### 2.1 Search strategy

Several studies were selected from various journals worldwide to present a brief review of different works on flood vulnerability using a geographic information system. For reviewing earlier published works, the systematic literature review was considered. Search string strategy has been used in five databases used for this review.

As mentioned in Table 1, including Francis & Taylor, Science Direct, Springer Link Sage Publication, and JSTOR database engine have been used to select studies and find different methods of resolving flood vulnerability and the role of geographic information system.

**Table 1:** Keywords search strings in the international database (2010–2020).

Source	String
Francis & Taylor, Science Direct, Springer Link Sage Publication, and JSTOR	TITLE-ABS-KEY Flood Vulnerability using geographic information system AND, GIS OR Climate Change, Geographic Information Systems, Floods, Vulnerability, Remote Sensing, Decision Making, Flooding, Flood Mitigation " Hazard, Flood Control, Disaster Management, Flood Planning, Hazard, Assessment, Mapping, Flood Preparedness, Spatial Analysis, Risk Management, Geographic Information System, Hydrological Modeling, Assessment Method, Disaster, Mapping Method, Hazard Management, Flood Recovery.

### 2.2 Screening process

In the five databases, the researcher only focused on the research articles. An extended list of different academic publications has been prepared and analysed to provide an insight into additional research on flood vulnerability. Keyword's analysis involved flood, vulnerability, flood vulnerability, flood vulnerability assessment, flood vulnerability assessment & geographic information system. SCI journals related to flood and vulnerability for detailed keywords analysis were critically reviewed and analysed (Table 2). Chosen keywords were related to the techniques for flood vulnerability and geographic information system.

**Table 2:** Keywords, titles, and abstract identification and evaluation in the international database.

S.No	Journals database	Flood	Vulnerability	Flood Vulnerability	Flood vulnerability & Geographic Information system
1	Taylor and Francis journal	160,247	379,338	32,967	14025
2	Springer Link	83157	66753	11389	3093
3	Science Direct	244,408	490670	38489	16035
4	Sage Journals	39319	151077	9438	3856
5	JSTOR	399,417	467,693	48,784	15548

A list of keywords used in various journals indexed in the five database search engines (Taylor and Francis journal, https://www.tandfonline.com/, Springer Link, https://link.springer.com/, Science Direct, https://www.sciencedirect.com/, Sage Journals, https://journals.sagepub.com/, and JSTOR, https://www.jstor.org/). Articles from various periodicals related to vulnerability to flood were assessed and critically evaluated based on their methodology. The articles from January 2010 to December 2020 have been selected for review. The researcher has used the method of systematic literature review.

**Figure 2:**
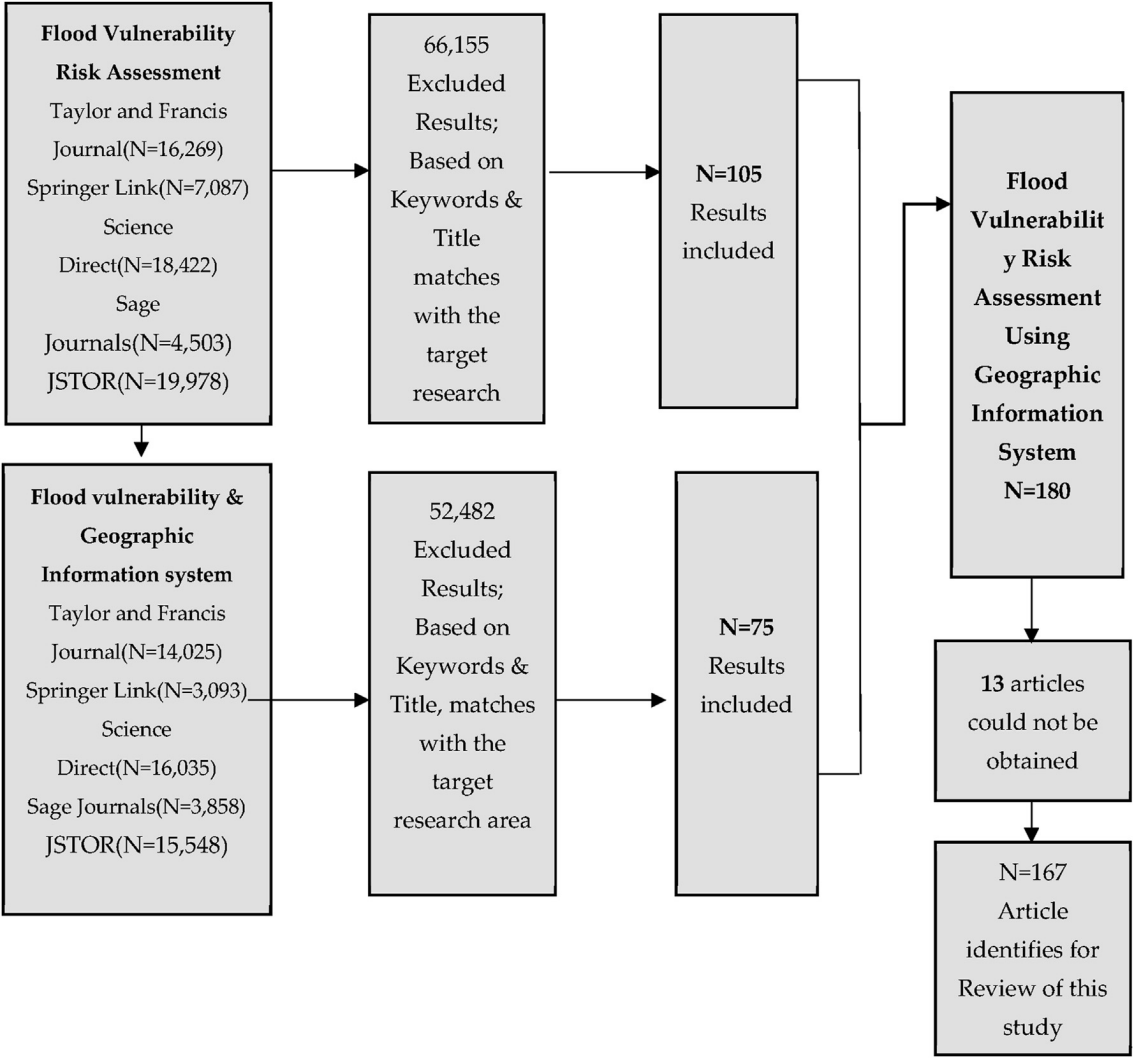
Stages of systematic literature review.

The second step is required to do the screening of the articles. Screening of the articles was based on keywords & title matches with the target research area, which is based on flood vulnerability and the role of geographic information system. The third step is to make the article eligible for review. In this research, 105 articles studies have been selected for flood vulnerability and, 75 papers have been chosen for the role of geographic information system. In the last stage of the systematic literature review, we provided the articles we used in the study. For this case, 180 studies have been finalised, but due to the limitation of the studies, 13 studies were not obtained. Therefore, for the final selection and to keep the quality appraisal high, the researcher has found 167 studies to be reviewed systematically, as demonstrated in Figure 2.

### 2.3 Inclusion/exclusion criteria

The flood vulnerability systematic review provides extra guidance on assessing the body of knowledge. The study also helps synthesise, formulate the available data, and improve overall data collection methodology. The systematic literature review consists of four critical steps, i.e. identification, screening, eligibility, and inclusion. The first step is the identification of the articles through different databases. For this review, the researcher has used five databases as discussed above in Tables 1 and 2. For identification through keywords and the title of the abstract, the researcher found 66,260 flood vulnerability results. For the keywords and abstract of flood vulnerability and geographic information system, 52,557 results were found in the initial review step. Included and exclusion criteria were developed after determining the relevant and applicable search terms, as shown in Table 3. The process of inclusion and exclusion involved all four stages, including identification, screening, eligibility, and inclusion, which helped us finalise the papers selected for the study. Along with the quality check, 167 articles were included in the review.

**Table 3:** Inclusion/exclusion criteria.

Inclusion criteria	Exclusion criteria
• Papers defined flood vulnerability, methodology and approaches.	• Papers that do not define flood vulnerability and their methods.
• Papers also investigated the flood vulnerability using geographic information system	
• Paper that defined social, physical environmental, and economical flood vulnerability.	• Papers that do not define approaches and methodology for flood vulnerability assessment
	• Papers that specify vulnerability to other natural hazards in the GIS context

## 3 Results

For decades, vulnerability emerged as a critical problem among scholars, primarily in the sense of natural disasters. Various authors have widely generalised the definition of vulnerability. Cutter and Adger have discussed crucial contributions in the event of vulnerability to natural hazards. Vulnerability assessment has been emphasised by Ratick, Brouwer, Revi, Kron, Brouwer, and Balica. In the United States, Cutter focused on vulnerability due to natural disasters, flooding, and cyclones. Flood vulnerability has been assessed by Kubal, Haase, Meyer, Messner, and Scheuer. In the sense of social insecurity, vulnerability at large was analysed. Flood has more interconnection with land conversion and climate change. Nonetheless, multiple studies have provided climate change weighting, especially for the induction of floods and consequent vulnerability in an area. Several statistical approaches, quantitative and qualitative techniques, and geospatial analysis have challenged the flood hazards in flood vulnerability assessment.

### 3.1 Concept of flood vulnerability

Researchers' perceptions of vulnerability have shifted over the last two decades, prompting many attempts to define and capture what the term means. After years, the International Panel on Climate Change defined vulnerability as "the extent to which climate change may damage or harm a system. It depends not only on a system's sensitivity but also on its ability to adapt to new climatic conditions," Watson et al. (1996) defined it as "the extent to which climate change may damage or harm a system."

Vulnerability, according to Blaikie et al. (2005), is an assessment of a person's or a group's exposure to the consequences of a threat, as well as their ability to recover from the event's impact. Green (2004) defines vulnerability as the possibility of harming a receptor. These latter three (quite similar) interpretations are current, and they define vulnerability as the risk of injury or harm. Kelly and Adger (2000) emphasise the need of seeing some vulnerability assessments as the endpoint of any appraisal, others as the focal point, and yet others as the beginning point. Finally, Van Der Veen and Logtmeijer (2005) expanded on the idea of vulnerability to explain flood vulnerability from an economic standpoint.

According to Gheorghe (2005), vulnerability is a function of sensitivity, resistance, and knowledge. Klein and Nicholls define environmental vulnerability as a consequence of three primary components: resistance, resilience, and susceptibility. Instead of narrowing the concept of vulnerability to elements at risk, exposure (damage potential) and (loss) susceptibility, Mitchell (2006) express vulnerability as a combination of exposure, resilience, and resistance. Adger (2006) defined vulnerability as the state of being vulnerable to harm due to exposure to stresses associated with environmental and societal change, as well as a lack of ability to adapt.

It is observed from the study of multiple experiments that (Chakraborty and Joshi, 2016) made the earliest attempt to describe vulnerability, stressing the need to explore the concept and the circumstances linked with vulnerability. He provided an extensive collection of concepts and models for determining vulnerability to help people recognise the idea of vulnerability, and flood vulnerability was described by Etinay et al. (2018) to understand the severity, extremes, and climate change. A substantial correlation between climate changeability and vulnerability was found by Fariza et al. (2018). In diverse settings, the degree of vulnerability and resistance varies. The scale has been discovered to be a practical component in deciding insecurity in a given area and performing a required study (Füssel, 2007) (Garbutt et al., 2015) (Fernandez et al., 2016) (Fatemi et al., 2017). From a timing viewpoint, it can be shown that the situations have changed over the duration to test vulnerable communities. Regarding the different techniques and models, such as the risk-hazard (RH) and hydrological models, is one of the most critical activities in determining flood vulnerability (Fernandez et al., 2016) (Hadi et al., 2017) (Hazarika et al., 2018), who analysed numerous studies from the 1980s, made an essential contribution to identifying and describing vulnerability.

The word "vulnerability" has been overused in science, particularly relative to climate change, leading to misunderstandings regarding what vulnerability means; any of the attempts proved to be partially successful in identifying risk in the sense of climate change. In science, however, vulnerability is frequently mixed with other syntaxes (Holand et al., 2011) (Horney, 2018) (Hoque et al., 2019). Work on exploring vulnerability, mainly concerning environmental hazards, was proposed by Etinay et al. (2018). Several authors, such as Cutter and Liverman, have provided a significant weak structure in their respective fields of work. According to (Holand et al., 2011) (Huang et al., 2012), the definition of vulnerability is similar to the concept of adaptability, sensitivity, frailty, resilience, and threat (Abid et al., 2021a, b, c).

During the time 2010–2020, the scholar looked at vulnerability concerning global climate change by taking into account the different factors such as risk, coping capability, and visibility (Huq and Hossain, 2015) (Islam et al., 2016). Vulnerability is described by Fatemi et al. (2017) as a condition in which people and places are at risk and which decreases their ability to respond to various environmental threats. Cutter suggested that vulnerability science involves an integrative approach to represent all elements, including environmental, social, and engineering processes and their dynamic interactions. In addition, risk differs spatially (the topography varies from place to place).

Therefore, it is essential to propose multiple solutions for different areas (Karagiorgos et al., 2016a, b). As a result, multiple evaluation approaches culminated in a complex system of methodologies, culminating in the proliferation of several variables that impact tolerance and resilience (Karmaoui et al., 2016). The disparity in the conceptualization of risk often stems from the distinct existence of academic work. The kind can see the difference in environments and locations. For example, in the case of natural disasters, risk varies based on the severity of the catastrophe. Etinay et al. (2018) proposed disasters as large-scale incidents originating from threats that significantly affect human civilisation. Floods, cyclones, hurricanes, and anthropogenic-induced events such as deforestation and industrial facility failures can be cited as examples. Coping ability corresponds to the ability to counteract or absorb results by adapting the need to determine climate sensitivity derives from an understanding of how individuals respond to different climatic environments (Brooks et al., 2005).

In large amounts of study, susceptibility to natural disasters has already been illustrated (Chakraborty and Joshi, 2016). In the case of geophysical risk, a lack of reliable data can often stymie vulnerability assessments. Furthermore, vulnerability is primarily related to disasters and is exacerbated by humans (Aroca-Jiménez et al., 2020). Aside from biophysical threats, the idea of risk must be formulated in a particular geographical sense. After vulnerability evaluation, the areas of acceptable danger and exposure to any natural disaster will quickly be established (Khamespanah et al., 2016). Khosravi et al. (2021) discussed environmental change vulnerability and the problems of adaptation and mitigation. He also stressed the implementation of climate change risk reduction strategies and various catastrophic events to decrease exposure and destruction.

### 3.2 Previous studies on flood vulnerability

The flood hazard risk implies people's or any region's exposure, sensitivity, and ceaselessness to flood threats and the failure to deal with their consequences (Lawal and Arokoyu, 2015). The need to understand the vulnerability of floods arises because of flood natural hazards assessment and evaluation, which will contribute to effective flood control and reduce its effect on different sectors of society (Lianxiao and Morimoto, 2019). Vulnerability is a phenomenon that emerged in the social sciences and is now becoming more common in disaster studies (Liu et al., 2021). The idea of vulnerability comprises numerous parts such as risk, exposure, and sensitivity and is multidimensional. There has been a considerable discussion among academics about the capacity to assess, assign and statistically quantify vulnerability among different classes over the last few decades (Lorente, 2019). In their works, numerous researchers have attempted to measure flood vulnerability. In addition, some associations still play a crucial role in empirically assessing flood vulnerability.

**Figure 3:**
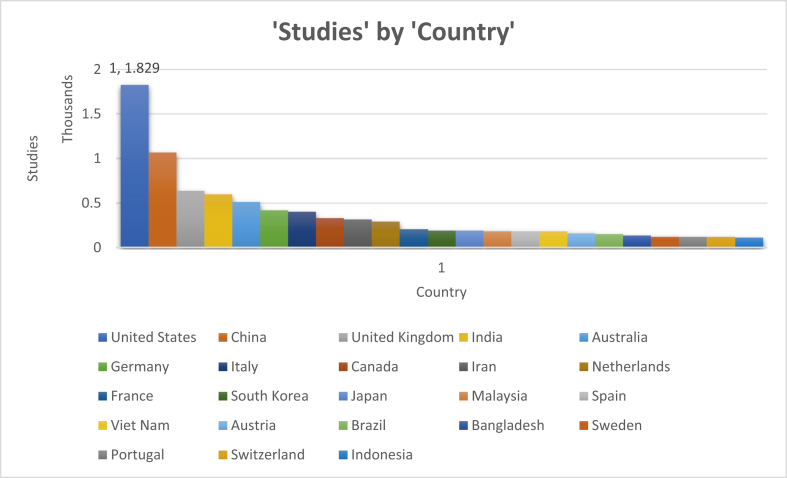
Distribution of the studies published by country (201–2020).

Lyu et al. (2018) stressed that ecological criteria should be taken into account, in addition to socioeconomic considerations, when discussing flood vulnerability in every area. The vulnerability of floods can be measured by classifying them into separate groups, such as natural, economic, and social vulnerability. Age, population density, impoverished settlements, and failure to access social resources can all be used to measure population exposure to flooding. Indicators such as degraded forests and land erosion may determine the environmental aspect. For social and economic elements, poverty, land resource base, and infrastructure usability may be considered (Ma et al., 2007). The flood assessment process should also include the cultural structure, gender, and economic systems; however, the concept of vulnerability has evolved over the last 30 years in the flood vulnerability index (FVI) (Mahato et al., 2021). The revised description also includes exposure, vulnerability, and resilience (Mahmood et al., 2017). In different areas of adaptation to a system, it has been applied. Figure 3 represents the distribution of the studies published by the country (2010–2020).

The definition of flood risk applies closely to the likelihood of high harm due to flood incidence and losses to natural, social, and economic conditions. The use of geographically based modelling to predict the probability of flood danger and flood damage is a deterministic approach to flood risk, contributing to flooding risk's economic effects in an area (Mohanty and Simonovic, 2021). The term vulnerability has been used in geography since the conceptualization proposed by Molloy et al. (2017). Flood vulnerability may be a combined risk and reaction outcome and generally decreases the population's health, contributing to hardship and inequality. In a flood threat, danger, reaction, and poverty are thus unrelated (Andrade and Szlafsztein, 2018).

### 3.3 Flood vulnerability in the context of underdeveloped countries

Floods have frequently wreaked havoc on developed countries. Around 7000 islands between the Philippines and Vietnam that are vulnerable to this threat, especially during monsoons, have been flooded for decades (Muqtada et al., 2014). Researchers have always been concerned with floods to tackle their effects and related vulnerability in developed countries. Many developed countries are at risk of floods due to various climate types related to severe weather and climate disasters such as monsoon rains and cyclones. Therefore, numerous hydrodynamic systems have been designed in these nations to study the complex existence of flooding. Somehow, due to the lack of hydrological and physiographical evidence, these models are reduced in number to answer and forecast floods in Asia, Africa, Bangladesh, and other developing countries (Islam et al., 2016). In their report, Mohanty and Simonovic (Mohanty and Simonovic, 2021) described flood vulnerability in the Lake Poyang area, estimating that about 55 percent of the region is at risk of flooding.

Especially in these countries, assessing physical and social deprivation is important because poverty is evident, and limited access to services. In the event of vulnerability, Molloy et al. (2017) sought to eliminate the difference between scientific and humanities studies by presenting physical, social, environmental, and economic aspects together as an integral component of vulnerability. For assessing physical vulnerability, they looked at infrastructure, construction architecture, and material content, as well as age, gender, health facilities, and emergency services for assessing social vulnerability. Another research by Muqtada et al. (2014) selected population, mortality, economy, and agriculture to examine the vulnerability of multi-dimensional floods using the tool of data envelopment analysis.

Vulnerability encompasses many risk factors, including social, environmental, physical, and economical. Therefore, these methods provide a more comprehensive, rapid, and reliable flood vulnerability assessment in a specific geographic region. Still, it is more difficult due to the lack of good data and the difficulty of quantifying many indicators, particularly social ones. As a result, the main limitation in this approach is that measurement of vulnerability must reflect social processes and material consequences, which appears complex and with many connections that are difficult to trace. As a result, exposure is difficult to reduce to a single indicator and difficult to calculate (Adger, 2006). On the other hand, computer-based modelling can estimate vulnerability at a local scale that is more sensitive than other methods since it takes into account unique local circumstances; yet, it has limited validity in data scarcity situations.

### 3.4 Different types of flood vulnerability

Floods have long been linked to extensive damage, not just to individuals and property but also to the climate. The vulnerability to flooding, cyclones, and climate change was illustrated by (Brooks et al., 2005). One of the most critical facets of risk is one's ecological footprint. They proposed that the essential metrics for evaluating environmental risk are resilience, functionality, and adaptation. According to Nasiri et al. (2016), climate change is a significant challenge to adjustment, resulting in social, economic, and environmental vulnerability. The term ecological vulnerability can be interpreted at different hierarchical stages, including the essence of the organism and its population, the species group, habitat form, and topography. The main components of environmental vulnerability are tolerance, preservation, and functionality (Neumayer and Plumper, 2007). Nguyen and Liou (2019) used a fuzzy interval-stochastic programming (MIFISP) model to test the efficacy of wetlands in minimising flood extent. In building the environmental vulnerability of wetlands, topography and hydrological features have a profound impact.

Various researchers analysed and measured societal exposure to environmental change and its resulting catastrophic actions (Ma et al., 2007) (Nguyen et al., 2020). The situations and instances under which people and multiple social and cultural communities respond to environmental change are essential for social adaptation. It is based on their economic activity and the existence of the wetlands. Economic vulnerability actively interferes with the wellbeing of livelihoods and the poverty of multiple social community groups (Ortiz et al., 2016) (Shivaparasad Sharma et al., 2018). Vulnerable populations may be defined as wage scarcity, resource inaccessibility, and social and economic crises (Balica et al., 2013). Mahmood et al. (2017) described flood vulnerability with social vulnerability in mind. They demonstrated more technological methods in flood risk management instead of relying on conventional hierarchical methods. Papathoma-Köhle et al. (2019) examined flood risk in low-income populations.

Intervention mechanisms for such communities are necessary to decrease the impact of the flood. Flood mapping, flood-vulnerable area boundary, and improved weather occurrence prediction are more straightforward with optical data (Brooks et al., 2005). In calculating flood depth, topographic models and flood vulnerability maps are necessary to help identify flood-prone areas (Percival and Teeuw, 2019). Fatemi et al. (2017) demonstrated the uncertain nature of researchers in using an analytical approach to hazardous waste disposal and reducing its implications on public health.

More psychiatric activity in women under the age of 65 was noticed in the study. Paprotny et al. (2020) made one of the central attempts to link flood susceptibility to human wellbeing, attempting to connect the mental disorder of Pennsylvania workers with flood using evidence obtained from personal interviews of respondents. An observational approach was introduced by Percival and Teeuw (2019) to research impairment in children (2–9 years) caused by flood events in Bangladesh between 2000 and 2020. The authors took a systematic approach to research flood hazards, risk exposure, and the associated posttraumatic stress.

### 3.5 Vulnerability assessment methods and a brief discussion on previous work

According to a survey of numerous studies, Kates (1971) suggested a decision model determine how individuals comprehend hazards, which was the first attempt to characterise vulnerability. The vulnerability was the name of the model. Birkmann and Wisner (2006) described vulnerability as a comprehensive and transdisciplinary concept. According to the study, indications and criteria for measuring vulnerability should be physically, economically, and socially related to the area of interest (Rashed and Weeks, 2003) (Syrbe and Walz, 2012). Balica et al. (2012) used indicators to demonstrate flood vulnerability. This indicator-based methodology for calculating Flood Vulnerability Index (FVI) has been approached differently for river basins, sub-catchments, metropolitan areas, and coastal floods (Adger, 2009). Rygel et al. (2006) proposed a composite vulnerability index for countries in the emerging stage and islands. The integrated vulnerability index for developing nations was created using available data. The findings suggested that small regions are more vulnerable than larger states (Dottori et al., 2018). Moss et al. (2010) chose ten representatives for each of the five climate responsiveness categories. These include sensitivity to the arrangement, food safety, human health awareness, ecosystem sensitivity, and water availability. These individuals were grouped to form sectoral indicators, responsiveness indicators, and coping or adaptive capacity indicators. They finally created climate change risk resilience indices based on these indicators (Lianxiao and Morimoto, 2019). da Silveira and Bonetti (2019) prepared a flood inundation map using advanced land imager (ALI) data and additional high-resolution microwave data, which was then employed in a flood vulnerability analysis. Because of their fast picture delivery (Feloni et al., 2020); (Kumar and Bhattacharjya, 2020) employed RADARSAT data, synthetic aperture radar (SAR), and Sentinel-1 & 2 to study flood hazard.

Blistanova et al. (2016) use GIS to assess the flood susceptibility of the Bodva river basin in eastern Slovakia based on various parameters. They used a variety of hydrological elements and geomorphological aspects of the basin, such as slope and soil type. These indications are assessed and included in the GIS to assign the study region to one of four vulnerability zones: acceptable, moderate, unpleasant, and unacceptable. Assess Addis Ababa's vulnerability in the Akaia catchment due to climate change and fast urbanisation. The peak discharge was calculated using the SWAT model, and the peak discharge was included as one of the indications. The General Circulation Models (GCM) data were used to forecast future rainfall, while Landsat pictures were used to create land use and land cover data. The findings demonstrate that climate change has caused a significant rise in discharge, which has increased vulnerability. Table 4 illustrates the previous works on different methodologies for assessing flood vulnerability.

**Table 4:** Different Methodologies for assessing flood vulnerability.

Type of Vulnerability	Methodology	References
Social Vulnerability	Indicator based approach, Weighted Sum Approach (WSA), Principal Component Analysis (PCA), and an Integrated Approach (IA), Interdependency analysis, indicator methodology, decision-making trial, method, Composite indicators approach, GIS-Based Multi-Criteria Approach Indicator based techniques using face to face interview, Analytic Hierarchy Process, A spatial vulnerability mapping approach, Indicator-based methodology incorporating Social Vulnerability Index (SoVI), Indicator based method, Spatiotemporal Analysis, Indicator-Based Approach, Analytical hierarchy process, Indicator-based approach, and the Delphi method	(Singh and Pandey, 2021), (Hosseini et al., 2021) (Nazeer and Bork, 2021), (Hussain et al., 2021) (Pathak et al., 2020), (Hoque et al., 2019) (Mavhura et al., 2017), (Terti et al., 2015) (Eidsvig et al., 2014), (Zhang, 2009)
Physical vulnerability	Interdependency analysis, indicator methodology, decision-making trial method., Indicator based approach, morphometric parameters were derived from SRTM DEM data using (GIS), Weighted Sum Approach (WSA), Principal Component Analysis (PCA), and an Integrated Approach (IA), GIS-Based Multi-Criteria Approach, Geospatial Indicator-Based Approach and Participatory Analytical Hierarchy Process, Flood generating factors: slope, elevation, land use/land cover, drainage density, rainfall, and soil types were rated and collected to mark out flood vulnerability zones using (GIS), Regression and GIS conditioning factors include digital elevation model (DEM), Pearson's correlation, multicollinearity, and heteroscedasticity analyses	(Singh and Pandey, 2021), (Hosseini et al., 2021), (Nazeer and Bork, 2021), (Hussain et al., 2021), (Vignesh et al., 2021), (Usman Kaoje et al., 2021), (Desalegn and Mulu, 2021), (Usman Kaoje et al., 2021), (Sami et al., 2020), (D'Ayala et al., 2020), (Chuang et al., 2020), (Yin et al., 2019), (Hoque et al., 2019), (Sahana and Sajjad, 2019), Hübl et al., 2016), (Al-Juaidi et al., 2018), (Hazarika et al., 2018), (Walliman et al., 2012) and, (Mehebub et al., 2015)
Environmental Vulnerability	Multicriteria evaluation in (GIS) to achieve the community-based assessment, The methodology is based on a mathematical index & The Flood Intensity Index, Digital map (to calculate mean elevation, slope, proximity to lagoon, sea, and drain length by area), Indicator-Based Approach, Analytical hierarchy process, Digital elevation model (DEM), indicator-based approach and Geospatial technique. 1:50,000 topographic map used. Six indices were included, And GIS data layers used	(Hazarika et al., 2018) (Dottori et al., 2016) (Codjoe and Afuduo, 2015) (Eidsvig et al., 2014) (Ma et al., 2007)
Economic vulnerability	Composite indicators approach, GIS-Based Multi-Criteria Approach, Flood generating factors: slope, elevation, land use/land cover, drainage density, rainfall, and soil types were rated and collected to mark out flood vulnerability zones using (GIS), Indicator-based approach, Numerical prediction, Gumbel Extreme Value Distribution Function, and information diffusion. Combining the fuzzy comprehensive evaluation method and the Delphi method, Composite indicators approach	(Nazeer and Bork, 2021) (Hussain et al., 2021) (Desalegn and Mulu, 2021) (Zhang, 2009) (Nazeer and Bork, 2021)

## 4 Discussion

Flooding and its effects have been taken into consideration in past reports. Under an academic context, flooding and its risk were studied separately. Previous research highlighted the vulnerability in terms of damages incurred by environmental disasters. A review of the five-database showed that more than 8000 academic papers are essentially dealing with flooding (Tables 1 and 2). The research has mainly concentrated on psychological, environmental, and economic insecurity. Current analysis on flood vulnerability shows the use of more effective techniques and strategies to measure the sensitivity of areas or persons to flooding (Ma et al., 2007) (Sulaiman et al., 2019) (Sulaiman et al., 2020a, b). In identifying flood susceptibility over space and time, a vague collection was considered necessary. Flood hazard forecasting focused extensively on disaster modelling, hydraulic modelling, flood emergence inspection, and multi-criteria techniques (Timmerman, 1981) (Tobin and Montz, 2004).

Predictors are valuable instruments for measuring flood vulnerability, the research has been checked, and an inventory of those indicators should be used for additional research Fernandez et al. (2016), Have published a comprehensive analysis of studies on social vulnerability and flood vulnerability undertaken around the world and an overview of different measures used to measure vulnerability. Geospatial tools such as GIS analysis and remote sensing techniques will be more beneficial (Rosales et al., 2021).

The keywords used in the review also suggested that fewer studies have been found on geospatial methods in flood risk analysis. It also indicated that flood vulnerability using geographic information system prediction would become more beneficial. A wide variety of work is being conducted worldwide on flooding and its effects on civilization. Main parameters for flood susceptibility have been found for coping capability and resistance. After reviewing numerous studies on flooding and vulnerability, it was discovered that various flood analyses had been in use for decades. However, the advent of remote sensing and GIS in flood assessment has provided more importance to flood analysis. Scholarly works on flooding were found to be complex.

In a single model, different vulnerability elements can be tested together. Social vulnerability is specifically connected to the failure of any party or society to deal with the repercussions of any occurrence (Wahab and Muhamad Ludin, 2018) (Wang et al., 2019) (Vignesh et al., 2021). The poorer part of the population is more vulnerable to flooding and other natural disasters. Fatemi et al. (2017) used a place threat model to examine social vulnerability, taking into account all facets of vulnerability and biophysical causes, possible danger, and intervention. Former efforts were outlined to extend the definition of flood risk in the sense of environmental disasters by using the moves framework (Sperotto et al., 2016) (Sayers et al., 2018) (Rehman et al., 2019).

Researchers have analysed flood susceptibility using different approaches and techniques, and further expansion is needed (Sayers et al., 2018). Geospatial instruments and statistical methods should be used to assess flood and vulnerability in the areas affected (Scheuer et al., 2011) (Shirazi et al., 2012) (Shariff and Hamidi, 2019) (Sulaiman et al., 2020a, b) (Abid et al., 2021a, b, c). These methods include a realistic flood assessment, particularly for those concerned about the scarcity of resources. Several global flood assessment models, such as a Global Flood Awareness System (GLFAS), are separate from political and social restrictions in partnership with different organisations. Such coordination can also be beneficial for better visualising flooding in the incidence and can quickly distinguish vulnerable areas. The earth is getting more urbanized, and the susceptibility to urban flooding has been considered in previous studies. Other types, such as storm waves, rainstorms, and rural flooding, require empirical research using parametric methods (Adger et al., 2005) (Huang et al., 2012) (Fernandez et al., 2016) (Rehman et al., 2019).

## 5 Conclusions

The following conclusions are drawn from a study of flood vulnerability assessment methods:

Since the 2000s, the current research has discussed multiple dimensions, strategies, and flood techniques and their vulnerability evaluation. Over 150 papers by the most cited researchers were carefully analysed to produce a sound and consistent study of different approaches. The behaviors of flood susceptibility evaluation were characterised by graphical representation of keywords that described methods and critical datasets and documentation of flood-related research. Advances in the methodological context and flood vulnerability evaluation frameworks were analysed, stressing the latest models used. A database of widely used flood vulnerability measures, approaches and techniques was analysed. The results showed that researchers were most interested in flash flooding, tidal floods, and urban floods.

GIS-based mapping, remote sensing imagery (RSI) are the tools and models used by scholars to determine flood hazard vulnerability. By broadening the definition of flood risk, differences between strategies and methodologies may be eliminated. Methods based on indicators were given a vital role in assessing vulnerability. However many researchers commonly use the indicator-based approach, but there are some challenges and complications related to weighting, aggregation, and standardization methods.

Conclude, numerous scholars are actively using Geographic information systems, various statistical analyses, Remote Sensing, and computer languages to conduct in-depth assessments of flood susceptibility. In this work, we tried to concentrate on ancient and novel data sources, spatial variables, and indicator-based technologies used to map the degree of vulnerability around the world. The main limitations of this study were the wide range of approaches used, the type of vulnerability studied, the number of references examined, and the selective focus of most studies on a single danger, namely flood. Nevertheless, the findings of this study identified several gaps that may be bridged by the creation of a new comprehensive vulnerability assessment system. Physical, social, environmental, and economic vulnerability indicators should all be considered in the suggested integrated framework, which should be internationally relevant for all sorts of disasters.

## Declarations

### Author contribution statement

All authors listed have significantly contributed to the development and the writing of this article.

### Funding statement

This work was supported by the TIER 1 project grant for: Modelling Spatial Flood Vulnerability Assessment using Geographic Information System for Disaster Risk Reduction in Sarawak (Project Number: H949).

### Data availability statement

Data included in article/supplementary material/referenced in article.

### Declaration of interests statement

The authors declare no conflict of interest.

### Additional information

No additional information is available for this paper.
